# Species Composition and Diversity of Parasitoids and Hyper-Parasitoids in Different Wheat Agro-Farming Systems

**DOI:** 10.1673/031.013.16201

**Published:** 2013-12-30

**Authors:** Zi-hua Zhao, Jun-He Liu, Da-Han He, Xiao-qin Guan, Wen-Hui Liu

**Affiliations:** 1 Agricultural School, Ningxia University, Yinchuan 750021, China; 2 Department of Biological Engineering, Huanghuai University, Zhumadian 463000, China

**Keywords:** cereal aphids, landscape structure

## Abstract

Insect communities depend on both their local environment and features of the surrounding habitats. Diverse plant communities may enhance the abundance and species diversity of local natural enemies, which is possible due to a higher abundance and species diversity in complex landscapes. This hypothesis was tested using cereal aphid parasitoids and hyper-parasitoids by comparing 18 spring wheat fields, *Triticum aestivum* L. (Poales: Poaceae), in structurally-complex landscapes (dominated by semi-natural habitat, > 50%, n = 9) and structurally-simple landscapes dominated by arable landscape (dominated by crop land, > 80%, n = 9). The agricultural landscape structure had significant effects on the number of parasitoid and hyper-parasitoid species, as 26 species (17 parasitoids and 9 hyper-parasitoids) were found in the complex landscapes and 21 were found in the simple landscapes (14 parasitoids and 7 hyper-parasitoids). Twenty-one species occurred in both landscape types, including 14 parasitoids and 7 hyper-parasitoids species. The species diversity of parasitoids and hyper-parasitoids were significantly different between the complex and simple landscapes. In addition, arable fields in structurally-simple agricultural landscapes with little semi-natural habitats could support a lower diversity of cereal aphid parasitoids and hyper-parasitoids than structurally-complex landscapes. These findings suggest that cereal aphid parasitoids and hyper-parasitoids need to find necessary resources in structurally-complex landscapes, and generalizations are made concerning the relationship between landscape composition and biodiversity in agricultural landscapes. Overall, abundance, species richness, and species diversity increased with increasing plant diversity and landscape complexity in spring wheat fields and increasing amounts of semi-natural habitats in the surrounding landscape.

## Introduction

Landscape structure plays an important role in species composition and diversity of insects (Thies et al. 2005). Insect communities depend on both the local environment and features of the surrounding habitats (Schmidt et al. 2005, 2008; Thies et al. 2008). Diversified plant communities are characterized by high properties of semi-natural habitats such as field margins, hedges, woodlands, and grasslands (Brewer et al. 2008; Anjum-Zubair et al. 2010). Several studies have shown that increasing landscape complexity can enhance natural enemy biodiversity in agro-farmland and thereby may support important ecosystem services such as biological control. Landscape patterns often affect ecosystem processes, such as species diversity and composition (Menalled et al. 1999; Roschewitz et al. 2005; Schmidt et al. 2008). A link between landscape composition and the local biodiversity in agrofarmland has been studied for many natural enemies in insects. Edge effects and spillover effects of species across non-crop-crop interfaces may result in increased abundance and species diversity in crops located in structurally-complex landscapes compared to structurally-simple landscapes (Zaller et al. 2008; Vollhardt et al. 2008). According to Tscharntke et al. (2007), more than 60% of the insect species found in an agricultural plot are likely to depend on the availability of semi-natural habitats. However, there are also studies that did not find a positive effect of landscapes complexity on abundance and species diversity. The insurance hypothesis predicts that high species diversity or functional plant groups stabilize ecosystem processes because the resulting functional redundancy allows a diversity of responses to agricultural landscape change and reorganizations following disturbance. Thies et al. (2008) have found that landscape complexity had no effects on species composition and diversity of cereal aphid parasitoids. But Menalled et al. (1999) obtained opposite results. The species in high trophic levels are more easily affected by landscape composition than species in low trophic levels. Hyper-parasitoids have not been studied in wheat fields.

The two most common aphid species in spring wheat fields, *Triticum aestivum* L. (Poales: Poaceae), in Yinchuan plain, Ningxia Hui Autonomous Region, Northwest China, are *Sitobion avenae* (Fabricius) (Hemiptera: Aphididae) and *Schizaphis graminum* (Rondani). They are mainly attacked by a range of cereal aphid parasitoids (Hymenoptera: Aphidiinae), which are attacked by a range of hyper-parasitoids (Hymenoptera: Pteromalidae: Encyrtidae). These parasitoids are solitary endoparasites and can act as important biological control agents in agro-farming systems (Melnychuk et al. 2003; Zhao et al. 2012). Their abundances and species diversity can vary between different landscapes, especially for hyper-parasitoids, but little is known about their diversity in relation to landscape complexity (Thies et al. 2008; Wackers et al. 2008). In this study, the diversity of aphid parasitoids and hyper-parasitoids was studied in spring wheat fields in structurally-complex and simple landscapes. Structurally complex-landscapes should support a higher diversity of cereal aphid parasitoids compared to structurally-simple landscapes because they should provide 1) a higher abundance and species diversity of alternative hosts due to a diverse plant community, 2) a higher amount of potential resource availability for parasitoids and hyperparasitoids (nectar and pollen), 3) more shelter from disturbances by agricultural practices, and 4) more potential overwintering condition due to a higher proportion of semi-natural habitats.

## Materials and Methods

The study was performed around the city of Yinchuan, Ningxia Hui Autonomous Region, Northwest China, in 2009–2011. Communities of cereal aphid parasitoids and hyper-parasitoids were studied in conventionally managed spring wheat fields in 18 agricultural landscapes that were located in the Yinchuan plain. Half of the fields were located in a structurally-simple landscape (n = 9), and the other half were in a structurally-complex landscape (n = 9). Landscape complexity was quantified using the percentage of arable land in a radius of 500 m around each field. This scale has been shown to be appropriate to measure effects of landscape complexity on cereal aphid parasitoids (Zhao et al. 2013a; Rösch et al. 2013). The percentage of arable land ranged between 87.3 ± 17.4% in the simple landscape and 38.4 ± 7.9% in the complex landscape. The latter landscape pattern typically contained more field margins, hedges, woodlands, and grasslands.

Parasitoids and hyper-parasitoids were sampled in wheat fields using sweep netting and hand collection in May–July (Thies et al. 2008). In each field, one transect ran through the field center, and one transect ran parallel to the field edge, therefore weed species could not totally be excluded from the sweep netting at the field edge. The transect through the field center started and ended a few meters from the edges to prevent edge effects and spill-over effects. Sampling consisted of walking the transect a single time while sweeping the top of vegetation at both sides. The sweep netting was carried out on warm and mostly sunny days and not after rain. To exclude possible effects of diurnal activity of the different species, sampling was conducted at different times of the day at four dates per field in July. Additionally, aphids were counted per 100 straws in the field centers on two dates (June and July) in pesticide and herbicide free areas ranging from 12 m × 10 m to 24 m × 20 m, depending on machine track disturbances, to show the undisturbed aphid population growth in the two different landscape types. All adult parasitoids and hyper-parasitoids were identified to species according to identification features.

The species density of the parasitoid samples from the field and the estimated number of parasitoid species were calculated using the jack-knife technique and were highly correlated (Spearman's rank correlation, r = 0.80, *p* = 0.002), thereby showing little sensitivity for sampling effects. Therefore, the raw data were used to calculate the Shannon-Wiener index for parasitoid species richness in structurally-simple and complex landscapes. Parasitoid density and species richness were converted to density and richness per 10 nets. Differences in aphid density, parasitoid density, and species diversity of parasitoids and hyper-parasitoids between the two agricultural landscapes types were tested using the Kruskal-Wallis test (Thies et al. 2008). The data per wheat field were merged for the statistical analysis. All statistical analysis was performed using SAS 6.1.2 (SAS Institute Inc. 1997).

**Figure 1.**
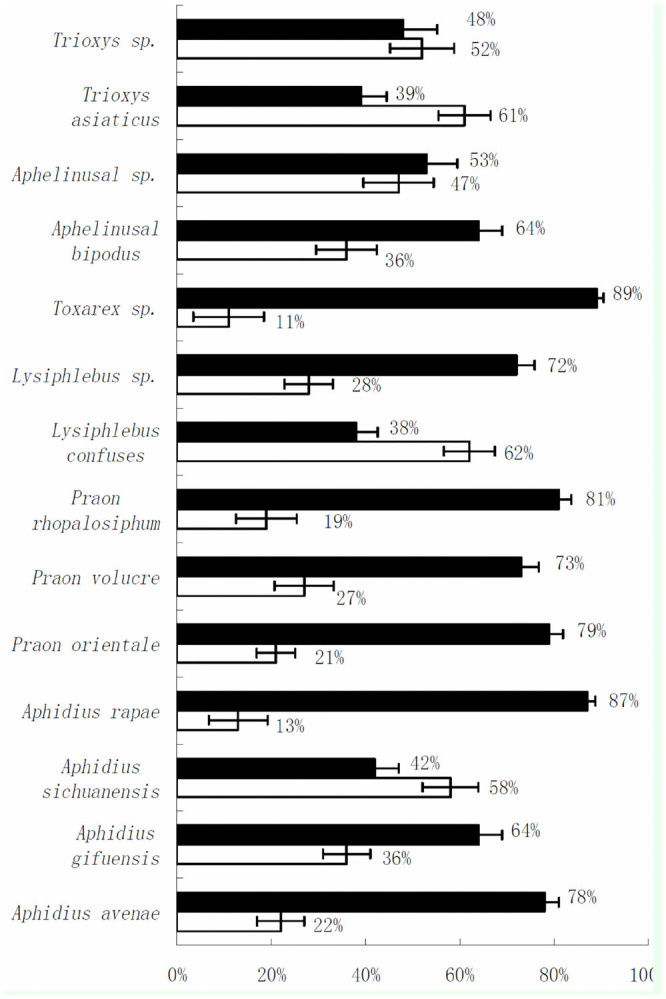
Distributions and species composition of cereal aphid parasitoids in simple and complex landscape in Northwest China during 2009–2011. Black boxes: complex landscapes; empty boxes: simple landscapes. Only species that simultaneously occurred in two patterns of agricultural landscapes are listed. High quality figures are available online.

**Table 1.**
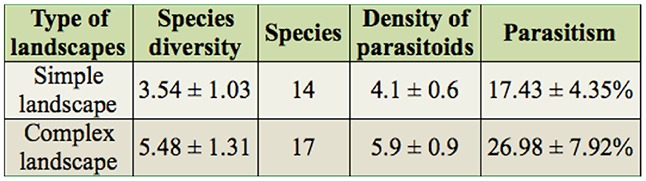
The ANOVA analysis of diversity, species, density, and parasitism of parasitoids in simple and complex landscape in Northwest China during 2009–2011.

## Results

In total, 7,305 parasitoids of 17 species and 4,324 hyper-parasitoids of nine species were collected in 18 wheat fields in the experiments. The total density of parasitoid individuals per 10 nets and the species diversity of total parasitoids were significantly different between the structurally-complex and simple landscapes (total density: *p* = 0.004; diversity: *p* = 0.023) (Table 1). In nine parasitoid species, *Aphidius avenae* Haliday (Hymenoptera: Braconidae), *Aphidius gifuensis* Ashmead, *Aphidius rapae* M'Intosh, *Praon orientale* Stary and Schlinger, *Praon volucre* (Halida), *Praon rhopalosiphum* Takada, *Lysiphlebus* sp., *Toxarex* sp., and *Aphelinus albipodus* Hay at and Fatima (Aphelinidae), density was higher in the complex landscape than in the simple landscape (Figure 1).

**Figure 2.**
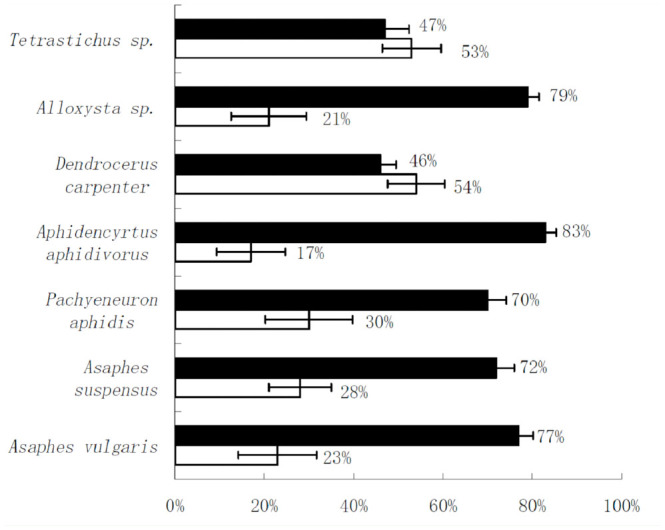
Distributions and species composition of cereal aphid hyper-parasitoids in simple and complex landscape in Northwest China during 2009–2011. Black boxes: complex landscapes; empty boxes: simple landscapes. High quality figures are available online.

The density and the species diversity of cereal aphid hyper-parasitoids were significantly different between the structurally-complex and the simple landscape (total density: *p* = 0.012; diversity: *p* = 0.018) (Table 2). The density of five hyper-parasitoids species, *Allosysta* sp., *Aphidencyrtus aphidivorus* (Mayr) (Hymenoptera: Encyrtidae), *Pachyneuron aphidis* (Bouché) (Pteromalidae), *Asaphes suspensas* Nees, and *Asaphes vulgaris* Walker, was higher in the complex landscape than in the structurally-simple landscape (Figure 2).

**Table 2.**
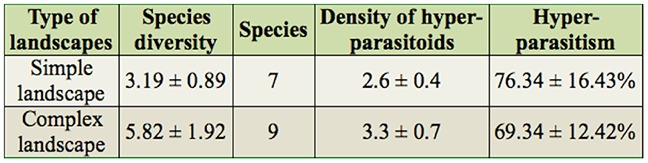
The ANOVA analysis of diversity, species, density, and parasitism of hyper-parasitoids in simple and complex landscape in Northwest China during 2009–2011.

## Discussion

The results of this study show that structural-ly-complex and structurally-simple landscapes supported a different species diversity and density of cereal aphid parasitoids. Thus the hypothesis that structurally-complex landscapes with a high proportion of semi-natural habitats support a higher species diversity and abundance of parasitoids and hyper-parasitoids than structurally-simple landscapes by providing alternative hosts, food, and overwintering conditions is supported by our study (Leslie et al. 2009; Garratt et al. 2010; Gagic et al. 2011).

Parasitoids and hyper-parasitoids appear to have profited from the high availability of semi-natural habitats in structurally-complex landscape. Spatial configuration and composition of landscape and habitat diversity have been shown to affect the abundance and species diversity of insects (Roschewitz et al. 2005; Zhao et al. 2013b). The species richness of generalist predators, such as carabid beetles and spiders, has been shown to be higher in wheat fields in structurally-complex landscapes than in simple ones (Schmidt et al. 2005, 2008). In the studied complex landscape, a higher species diversity of plants was found and should therefore provide a higher diversity of plant-associated potential hosts for cereal aphid parasitoids and hyper-parasitoids. Additionally, complex landscapes with a high percentage of semi-natural habitats could also enhance the activity of hyper-parasitoids, which had a negative effect on primary parasitoids of cereal aphids (Thies et al. 2008). However, some studies obtained a contrasting result, finding that the diversity and abundance of species was relatively the same in both structurally-complex and simple landscapes. Parasitoids and hyper-parasitoids may be more sensitive to habitat complexity in agricultural landscapes than other types of natural enemies. But, some studies did not find significant differences in species composition and diversity of parasitoids between structurally-complex and simple landscapes (Thies et al. 2005, 2008; Vollhardt et al. 2008). In our experiment, the structurally-complex landscape showed a higher species diversity and abundance of parasitoids and hyper-parasitoids compared with the simple landscape.

We summarize three points to interpret the results in our research and literature (Brewer et al. 2008; Tscharntke et al. 2007; Zhao et al. 2012). The first, parasitoids and hyperparasitoids are relatively oligophagous rather than generalist predators, and they need migrate between different habitats to search for a host in wheat habitats and nectar as a food source in semi-natural habitats. Parasitoids and hyper-parasitoids of cereal aphids seem to be spatially and temporally closely-linked with specific plant species, given that complex landscapes typically provide abundant resources. Structurally-complex landscapes have higher plant diversity, and thus more nectar and pollen as food, and can therefore support higher species diversity and abundance of parasitoids and hyper-parasitoids. Second, parasitoids and hyper-parasitoids always require alternative hosts in complex landscapes with multiple habitat patches as overwintering conditions. In general, seminatural habitats support more alternative host plants, and therefore may provide more amounts of alternative hosts for adult parasitoids and hyper-parasitoids. Hence, both parasitoids and hyper-parasitoids in different agro-farming systems are promoted to various habitats depending on landscape composition. Third, the assemblage and structure of plant species might play an important role. Only some plant species that supply specific resources for natural enemies could enhance biological control of the pests. But, many plant species could not supply any resources for parasitoids in seminatural habitats, which would lead to the same diversity of parasitoids and hyperparasitoids in both complex and simple landscapes. Landis et al. (2000) found that 165 plant species could have an important function in biological control after screening for thousand of plant species, and suggested specialist plant species can supply shelter, alternative hosts, and an overwintering site, while generalist plant species can not. These plant species had a functional plant pattern. The assemblage and composition of functional plant patterns may play an important role in biological control in agricultural landscapes (Thomson et al. 2010; Perdikis et al. 2011). Structurally complex landscapes that had these functional plant patterns could show higher species diversity of insects compared with simple landscapes (Zhao et al. 2013b).

Agricultural policy should take the surrounding landscape composition into account when considering using semi-natural habitats to enhance species diversity and abundance of natural enemies, and their biological control function, in arable fields (Schmidt et al. 2008; Haenke et al. 2009). Creating locally semi-natural habitats is more effective in structurally-simple landscapes containing a high percentage of arable fields, while in complex landscape, keeping high species diversity and abundance of natural enemies is important (Garratt et al. 2010; Zhao et al. 2012).
